# Solution Blow-Spun Poly (Ethylene Oxide)-Polysulfone Bicomponent Fibers—Characterization of Morphology, Structure, and Properties

**DOI:** 10.3390/polym15163402

**Published:** 2023-08-14

**Authors:** José Ernesto Domínguez-Herrera, Octavio Maldonado-Saavedra, José Roberto Grande-Ramírez, Luis Rolando Guarneros-Nolasco, Javier González-Benito

**Affiliations:** 1 Department of Nanotechnology, Universidad Tecnológica del Centro de Veracruz, Cuitláhuac 94910, Veracruz, Mexico; oct206@hotmail.com; 2 Department of Metal-Mechanic, Universidad Tecnológica del Centro de Veracruz, Cuitláhuac 94910, Veracruz, Mexico; jose.grande@utcv.edu.mx; 3 Department of Informatic, Universidad Tecnológica del Centro de Veracruz, Cuitláhuac 94910, Veracruz, Mexico; luis.guarneros@utcv.edu.mx; 4 Department of Materials Science and Engineering and Chemical Engineering, Universidad Carlos III de Madrid, 28911 Getafe, Spain; javid@ing.uc3m.es

**Keywords:** core-shell fibers, solution blow spinning, morphology, polysulfone, polyethylene oxide, bicomponent fiber

## Abstract

Solution blow spinning was used to prepare nonwoven bicomponent fibers constituted by poly (ethylene oxide)-Polysulfone (PEO-PSF). As a new material, deep characterization was carried out to have a database to understand final performance regarding its multiple functions as a potential material for biomedical applications. The morphology was studied by field emission scanning electron and transmission electron microscopy and optical profilometry. Structural characterization was carried out by Fourier transform infrared spectroscopy and thermal degradation by thermogravimetric analysis. Additionally, wettability and mechanical behavior were studied by contact angle measurements and tensile tests, respectively. The bicomponent material was constituted of fibers with a structure mainly described by a core-shell structure, where the PSF phase is located at the center of the fibers, and the PEO phase is mainly located at the outer parts of the fibers, leading to a kind of shell wall. The study of possible interactions between different phases revealed them to be lacking, pointing to the presence of an interface core/shell more than an interphase. The morphology and roughness of the bicomponent material improved its wettability when glycerol was tested. Indeed, its mechanical properties were enhanced due to the PSF core provided as reinforcement material.

## 1. Introduction

In recent decades, fibers with submicrometric diameters have attracted more and more interest from academics and industry due to the vast possibilities for their applications in several areas, such as the treatment of oil water, drug delivery, tissue engineering, manufacturing, biofunctionalization, and cell interactions [1,2]. Therefore, fibers with different morphologies in terms of shapes and sizes were studied because of the great effect of morphology and the available surface on final properties and performance.

There are several approaches to producing polymers constituted by fibers of small size: electrospinning [3], melt spinning [4,5], and centrifugal spinning [6], among others. However, solution blow spinning (SBS) has emerged lately because of its simplicity, reduced cost, relatively high rate of material production, and the possibility of dispensing the material in a specific location (in situ) [7,8,9].

Although the SBS technique is already well known, many modifications and studies have been made in order to understand the influence of processing conditions on fiber production [10,11]. As a result, currently, the research community has a better idea of how to tailor morphologies through the smart selection of processing conditions, thus inducing particular properties focused on achieving better performance in a particular application. For instance, Polysulfone and Poly (ethylene oxide) are attractive materials due to a blend of these materials used for gas diffusivity [12], ultrafiltration [13], and polymer electrolytes [14], among others.

Recently, more complex structured fibers have been receiving attention. Through the use of nozzles that allow one to obtain fibers of complex morphologies arising from the particular disposition of several polymer phases, new materials with improved or even new functions are being prepared. Among them, those constituted by an inner part (core) constituted by a phase enriched in a particular polymer and an outer part (shell) constituted by another phase enriched in another polymer are receiving special attention because of their potential as new systems for controlled release of active agents [15]. However, other configurations such as side-by-side (S/S) or islands in the sea (I/S), can be obtained [16,17,18]. In general, systems such as these can combine many capabilities from two or more components onto a single fiber, making multicomponent fibers appealing. Properties such as mechanical resistance, wettability, degradation, and others can be enhanced compared to mono-component fibers [19,20].

These systems are obtained mainly by electrospinning, but recently, a novel technique called SBS has been used to produce core-shell systems. Although hydrophilic and hydrophobic polymers fabricated by SBS have been used to control the release of compounds [21,22], other applications that enhance functionality, improve mechanical properties or compatibility and stability of materials have been little explored. This work aims to prepare bicomponent materials of Polysulfone/Polyethylene oxide constituted by core/shell fibers. Several properties, including wettability and mechanical behavior, will be studied and compared with fibrillar monocomponent materials.

## 2. Materials and Methods

### 2.1. Materials

Polysulfone (PSF) in the form of pellets (Mw = 35,000 g·mol^−1^ and Mn = 16,000 g·mol^−1^) and Poly (ethylene oxide) (PEO) in the form of powder (Mv = 100,000 g·mol^−1^) were used as the polymer components of the materials to be prepared. Chloroform (Ch) and acetone (Ac) were used as the solvents to prepare the corresponding solutions to be blow spun (purity higher than 99.5%). Sigma-Aldrich supplied polymers and solvents. All of the materials were used as received without further purification. Glycerol (supplied by Panreac) and distilled and deionized water (prepared in the UC3M laboratory) were used as the test liquids for contact angle measurements.

### 2.2. Sample Preparation

Materials were prepared by solution blow spinning (SBS), which requires a polymer solution injected in the inner channel of a concentric nozzle and the action of adequate air pressure at the exit of the nozzle to stretch the solution and favor evaporation of the solvent to produce fibers. To prepare the PSF solutions, 1 g of PSF was carefully weighed out and added to 8 mL of chloroform; then, the mixture was stirred until full dissolution. After this, 2 mL of acetone was poured into the solution, and the new mixture was subjected to vigorous mechanical stirring for 20 min. Therefore, PSF solutions of 10% *w*/*v* were prepared in Ch/Ac solvent mixture with a composition of 8:2 *v*/*v*. To prepare the PEO solutions, 1 g of PEO powder was weighed, added to 10 mL of chloroform, and stirred until full dissolution. Therefore, PEO solutions of 10% *w*/*v* were prepared in Ch solvent. Solutions were prepared at 28 °C and relative humidity of 26%.

**Figure 1:**
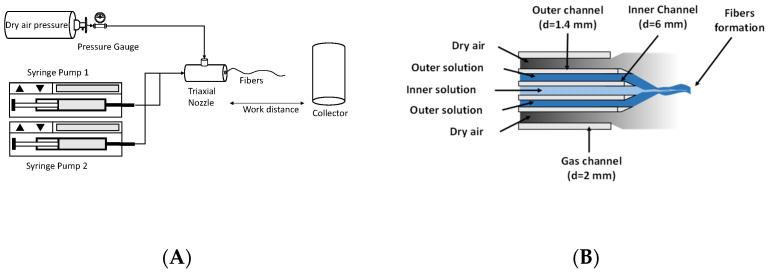
Schematic SBS setup (**A**) and triaxial nozzle (**B**) to produce bicomponent fibers.

The fibrous materials based on PEO and PSF were fabricated using a homemade device (Figure 1A) as already described in a previous article [16]. To prepare bicomponent fibers, a modified nozzle was used [17]. Figure 1B shows a scheme of the triaxial nozzle used to produce the bicomponent fibers. Three channels are observed; PSF and PEO are injected into the inner and outer channels, respectively, while pressurized air is passed through the outer channel. At the exit of the nozzle, the air exerts enough force to stretch the solutions and favor the evaporation of the solvents, leading to the formation of fibers with an expected coaxial structure that are finally collected on a rotating drum covered by aluminum foil located at a certain working distance (WD).

Nine- and fifteen-gauge needles were used for the inner and outer channels, respectively. The diameter of the gas channel was 2 mm. The conditions used to carry out the SBS process were characterized by the following parameters: flow rate or solution feeding rate (FR), 0.5 mL/min; air pressure (AP), 2 bars; working distance (WD), 20 cm; protrusion of the inner nozzle with respect to the outer nozzle, 1 mm; and rotation speed of the cylindrical collector, 200 RPM.

### 2.3. Instrumentation

The materials’ morphology was studied by field emission scanning electron and transmission electron microscopy (FESEM and FESTEM, respectively), using a field emission TENEO microscope (FEI). Additionally, EDSs for the materials were obtained. In the case of FESEM, an acceleration voltage of 5 kV was used, and the images were constructed with the signal arising from secondary electrons (SE) and detected with an Everhart Thornley detector. To avoid electrostatic charge accumulation over the materials, samples were gold-coated by sputtering using a Leica EM ACE 200 low vacuum coater with a current of 30 mA for 90 s. On the other hand, in order to better visualize the inner parts of the microfeatures of the materials, scanning transmission electron microscopy was used (FESTEM) with an acceleration voltage of 22 kV. In this case, the materials were collected directly during the SBS process on copper grids for proper observation by FESTEM.

The structure of the materials was studied by Fourier transform infrared spectroscopy (FTIR). Absorption spectra were obtained in the transmission mode within the range 600 to 1600 cm^−1^, with 32 scans per interferogram. Fibers were collected on KBr discs during the SBS process and then located in the infrared beam of the spectrometer (FT-IR Spectrum GX spectrophotometer, Perkin-Elmer).

A topographical study at the microscale of the materials was carried out by the use of an optical profilometer (Olympus dsx500). From the analysis of the 3D images obtained, roughness parameters were obtained using at least 10 linear profiles randomly taken from the images, and a 75.8 cut-off (λc) deduced from the UNE EN ISO 4288 standard, as described previously, was used [18].

Thermal degradation of the materials was studied by thermogravimetric analysis using a PerkinElmer Pyris 1 TGA thermogravimetric balance. The experiments were carried out on samples of about 10 mg by heating from 30 °C to 800 °C at a heating rate of 10 °C/min under a nitrogen atmosphere.

The wettability of the materials was studied from contact angle measurements based on the sessile drop method using an OCA-15 KR-SS GmbH tensiometer. Two test liquids (water and glycerol) were used to study the surface characteristics of the materials. In each case, the contact angle was taken as the average value from at least 10 measurements on 10 drops dispensed at distinct or separate locations on the surface of the materials.

The mechanical behavior of the fibrous materials was studied by performing conventional tensile tests using a universal testing machine (Microtest) equipped with a 15 N load cell. Specimens with rectangular geometry of 42 mm × 9 mm were tested. The thicknesses were determined through 4 measurements in different positions of the specimen to obtain a significant average value. In all cases, the gauge length was 20 mm. Each test was performed at a 10 mm/min speed at room conditions. Young’s modulus, tensile strength, and strain at break were determined from the stress–strain curves obtained from the tensile tests. Likewise, the values of said parameters were obtained as the average obtained from at least five tests carried out on five specimens of each material.

Apparent density ($\rho_{a}$) and porosity (∅) of the material were calculated using Equations (1) and (2), respectively.

(1) $$\rho_{a}=\frac{Mass}{area/thickness}$$

(2) $$\emptyset =\left(1-\frac{\rho_{a}}{\rho_{B}}\right)100\%$$

Density of bulk ($\rho_{B}$) for bicomponent material was calculated with Equation (3), where *F* and ρ correspond to fraction of volume and density of material, respectively.

(3) $$\rho_{B}=F_{PEO}\rho_{PEO}+F_{PSF}\rho_{PSF}$$

### 2.4. Data Analysis

The obtained images were analyzed with the free software ImageJ V.1.52a, US. Fiber orientation was evaluated using the OrientationJ plugin. Although this plugin was already used by other authors [19,20], in this article expanded explanation is given in order to understand the final analysis of the results better. Angle distribution was divided from −90° to +90°. Additionally, the average diameter was obtained by dividing the image into four quadrants, taking at least 50 random fibers chosen from each zone to finally have at least 200 fiber diameter measurements of each image obtained.

## 3. Results and Discussion

**Figure 2:**
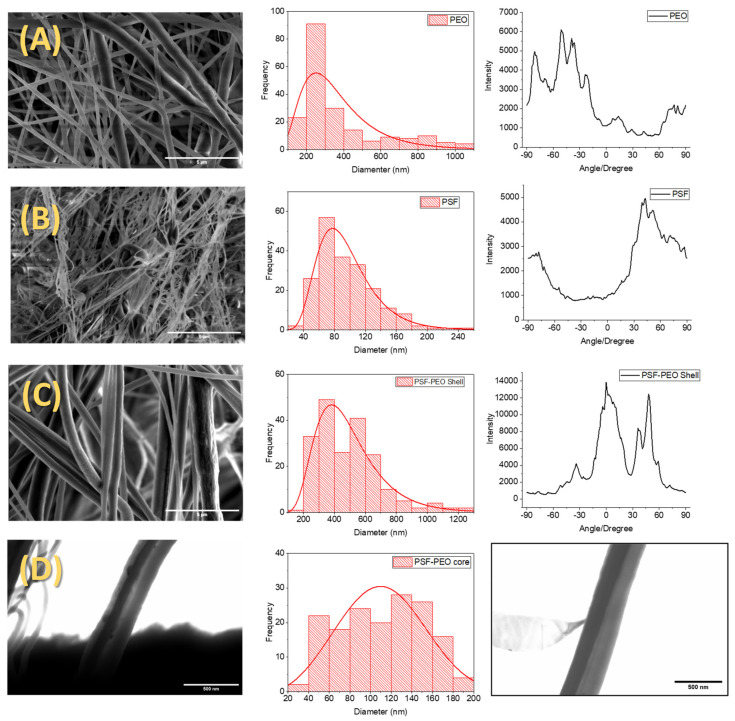
Images, distribution diameters, and fibrous orientation of materials obtained from PEO (**A**), PSF (**B**), bicomponent fibers (**C**) obtained with FESEM, and internal structure of bicomponent fibers (**D**) obtained with FESTEM.

Scanning electron microscopy was used to carry out a morphological study of the prepared materials in which the preferential orientation and sizes of the fibers in terms of their diameters were considered. Figure 2 shows some of the SEM images obtained for the materials obtained by SBS, the complex bicomponent system PSF-PEO, and the neat systems PSF and PEO for comparison. Moreover, the diameter distributions obtained from the corresponding image analysis are also shown. As can be seen, the materials were mainly constituted by fibers with an apparently cylindrical shape and high homogeneity in terms of size, as could be deduced from the diameter distributions. As reported, Anderson–Darling statistics were tested for normal, lognormal, Weibull, and Gamma fit [8]. In general, the fiber diameter distribution for the three materials presented a normal Log distribution. Because this distribution was not symmetric, the use of the average value was not considered significant for data treatment. Consequently, the median value was used to avoid the effects of extremes. It was observed how the size of fibers was highly dependent on the material used, being the thinnest of those obtained with PSF (~100 nm); the fibers of neat PEO presented a diameter of about 300 nm, and bicomponent fibers showed diameters of about 400 nm larger than those obtained from the neat polymers as expected, because the two components were added using a coaxial nozzle with larger exit diameter.

In particular, complete fibrillar morphology was observed on PEO material with a median diameter of 277 ± 70 nm (Figure 2A). PEO diameter fabricated with SBS was smaller than reported by others produced with a commercial airbrush [6]. Perhaps a stable region of low pressure was formed due to the controlled flow rate, causing a reduction in the fiber diameter as reported [21,22,23]. On the other hand, SBS fibers produced with the same concentration using a short working distance (15 cm) showed high diameters [24], which could be attributed to insufficient stretching of dissolution to the collector. Finally, SBS fibers with the same diameter were produced using a low concentration (6%) and short work distance (15 cm) [25]; the formation of fibers with the same diameter can be explained by the boiling point of the solvents used. Although the boiling point of dichloromethane is lower than that of chloroform/acetone, the evaporation rate of dichloromethane could explain the fiber formation in short work distances and low concentrations, allowing an adequate stretching of dissolution.

On the right side of Figure 2, orientation distribution plots are depicted to offer insight into the preferred orientation of fibers. It is observed that neat polyethylene oxide (PEO) exhibits a relatively wide distribution of possible orientations, displaying heterogeneity likely caused by the presence of hair-like protrusions emanating from the primary fiber body.

The materials prepared with PSF (Figure 2B) also present mainly a fibrillar structure, although material accumulation in the form of corpuscles can be observed. The formation of this microstructure is attributed to the use of low molecular weight PSF (Mw = 35,000) and a low concentration (10% *w*/*v*). When the concentration is low enough, some individual droplets can be ejected from the nozzle without being deformed to obtain fibers, forming these corpuscles upon reaching the collector [22]. The mean fiber diameter obtained in the case of SBS PSF was 85 nm, comparable to previously reported research about the production of PSF fibers under optimal SBS process conditions [8]. Compared with PEO material prepared by SBS in this work, PSF-based fibers showed more homogeneous distributions in terms of their orientations, although there was not a clear preferential orientation since the distribution widths were around 60°.

The SEM images of fabricated bicomponent material (PEO-PSF) also present a fibrillar morphology (Figure 2C), with hardly any microconstituents in the form of corpuscles being observed. For this material, two preferential orientations were obtained and shifted 45° to each other. One possible reason for this result might be the appearance of fibers with different distributions of the two phases, one symmetric and the other asymmetric. If perfect coaxial fibers are obtained, the weight is symmetrically distributed along the fibers. If PSF and PEO phases are heterogeneously distributed along the fibers, it is expected to be a deviation of the fiber trajectories to the collector due to the effect of gravity.

Observations were also made using scanning transmission electron microscopy (STEM) for the system based on PSF-PEO. The higher capacity of sulfur to scatter electrons should be useful to distinguish the PSF phase from the PEO phase; PSF is expected to have darker contrast in the image due to sulfur content. STEM images confirmed the bicomponent structure of PSF/PEO-based fibers from the observation of dark to clear contrasts. In principle, dark regions should correspond to PSF. After careful observation of many fibers, two main morphologies were visualized: (a) core-shell (C/S) morphology (Figure 2D left), with a dark region (PSF-rich phase) approximately in the center of the fiber and a cleared region at the outer part of the fibers (PEO phase), and (b) side-by-side (S/S) morphology (Figure 2D right), with the fiber divided into two regions, one on top or next to the other, depending on the direction of observation. C/S and S/S morphologies were confirmed by EDSs, due to C/S fibers present along the fiber peaks and intensity for neat PEO. In contrast, S/S morphology shows PEO and PSF peaks and intensities along the S/S fiber morphology. Perhaps vigorous bending after lineal dissolution stretching [26] causes a displacement of the PSF phase to the outer part of the PEO fiber. This hypothesis could be explained by entrapped core solvent inbound in a vapor phase. Core solvent evaporation can occur at both the shell and the menisci, causing displacement of the PSF phase to the PEO wall, forming a side-by-side structure [27].

From the STEM images, core and shell diameters were measured. The core corresponding to PSF and shell to PEO were 111 and 458 nm, respectively. The diameter of bicomponent fibers was higher than those of the individual PSF and PEO fibers. Conceivably, the convergence of two dissolutions at the nozzle tip causes an increase in the volume of material ejected—consequently, the diameter fiber increases.

Despite the concentration of PSF used to fabricate fibers by SBS seeming inadequate due to its low value, when PEO was added simultaneously through a triple concentric nozzle, PSF fibers without corpuscles were obtained. Consequently, using PEO as shell material stabilized the formation of PSF fibers, as reported for other materials [28]. In conclusion, the SBS process is advantageous compared to electrospinning; both solutions must meet the viscosity requirements (controlled by the solution concentration) to produce fibers [29].

**Figure 3:**
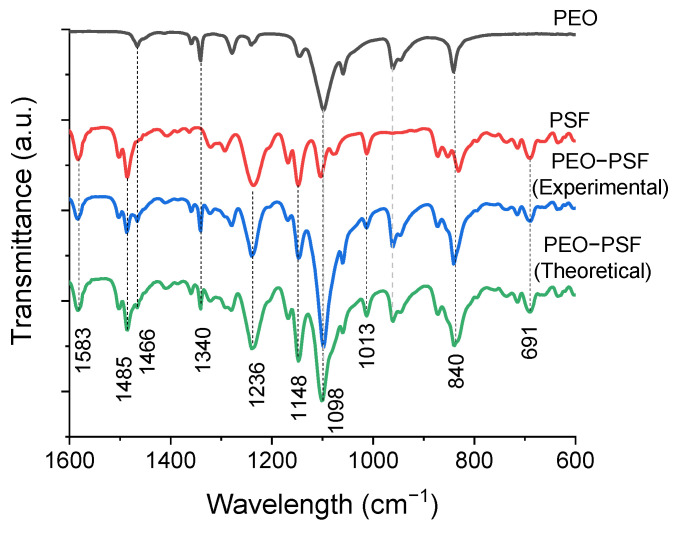
FTIR spectra of fibrous materials of PEO, PSF, and PEO-PSF.

The specific functional groups of the prepared materials were studied by FTIR. The fingerprint region of the polymeric materials under study is shown in Figure 3, where the peaks associated with the main absorption bands of the polymers are labeled.

The PEO spectrum (Figure 3) showed the presence of bands corresponding to C-O-C bond tensions at 1059 cm^−1^, 1098 cm^−1^, and 1148 cm^−1^ [30]. On the other hand, the peaks centered at 840 and 960 cm^−1^ were assigned to rocking vibrations of methylene CH_2_ groups. Furthermore, peaks at 1278 cm^−1^ and 1236 cm^−1^ are usually assigned to torsional movements of the CH_2_ group, while the peaks at 1340 cm^−1^ and 1361 cm^−1^ correspond to flutter movements of the CH_2_. Finally, the 1466 cm^−1^ peak was assigned to absorption due to activation of the CH_2_ group in the form of a scissor’s movement [25,31].

The infrared spectrum corresponding to PEO obtained by SBS under the conditions indicated in the experimental part coincides with that obtained for PEO using other SBS conditions [25]. In fact, PEO fibers produced with the same condition (concentration, blend of solvents, and production parameters) have been reported without FTIR spectrum alteration [24]. Therefore, it can be said that small variations in the SBS conditions do not lead to structural changes in the PEO.

On the other hand, PSF fibrous material prepared by SBS presented an IR spectrum (Figure 3) comparable to that obtained for PSF prepared by electrospinning [32]. Therefore, it can be concluded that both methods of preparation of materials lead to fibrous PSF-based materials with the same structure. Particularly, bands at 1013 cm^−1^ and 1103 cm^−1^ were observed, which are usually assigned to C-H bends in the aromatic carbons of the PSF [32]. In addition, at 1148 cm^−1^ and 1169 cm^−1^, a characteristic absorption peak of the symmetrical tension of the O=S=O group was detected; peaks at 1236 cm^−1^ are typically assigned to the symmetric strain of the C-O-C group; at 1292 cm^−1^ and 1322 cm^−1^, peaks associated with the asymmetric tension of the O=S=O group were also observed, and the peaks at 1488 cm^−1^ and 1586 cm^−1^ were assigned to the C=C tension of the aromatic ring [33,34].

Finally, the theoretical spectrum for PSF-PEO was inferred by adding the PEO spectrum to PSF directly. In this sense, it could be clear if new peaks in the PSF-PEO spectrum were detected. In the spectrum obtained for the PSF-PEO bicomponent fibers, no vibrational displacements, masking of the bands, or appreciable changes in the peaks corresponding to the pure polymers were observed. Furthermore, there was not any evidence of new bands. All of these observations demonstrated that there were no specific interactions between the polymers.

Therefore, it can be concluded that obtaining bicomponent fibers through the SBS technique using a coaxial nozzle in which each component is injected separately does not induce miscibility between the PEO and PSF polymers. Perhaps the appearance of the characteristic peaks of PEO and PSF in bicomponent fibers is due to the fraction of PSF fibers that are not completely centered. Therefore, a certain fraction of PSF fibers is outside the PEO Shell, forming a side-by-side structure due to vigorous bending after lineal dissolution stretching [35,36].

As previously stated, the STEM images clearly showed the limit between one phase and another in C/S fibers due to the difference in hue associated with each of the polymers because of different degrees of electron scattering. This result also reveals that there was no homogeneous mixture between the polymers, as the lack of specific interactions between the two polymers suggested.

Analysis of 3D images obtained by optical profilometry determined the roughness parameters R_a_, R_y_, and S_m_ (Table 1).

**Figure 4:**
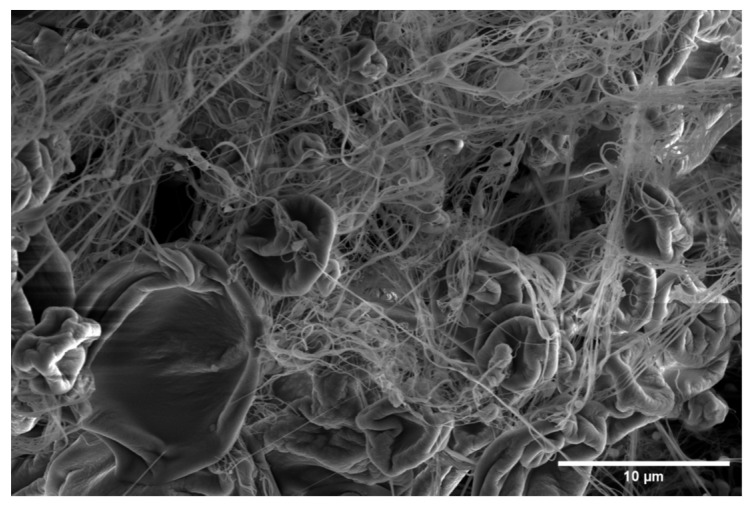
Representative SEM image of SBS PSF material showing typical microconstituents found.

Although all materials under study presented similar roughness identified by R_a_ and R_y_, the PSF material had a clearly higher value of S_m_, which meant that it had larger size heterogeneities that could be associated with the presence of bumps and regions with an accumulation of material, as can be seen in Figure 4. Although the PSF materials were constituted by fibers with clearly smaller diameters compared to PEO and PEO-PSF, mean spacing of profile irregularities was 94% higher. Perhaps this parameter increased because of additional surface heterogeneity due to the presence of other types of microconstituents, such as beads or corpuscles, on the surface of the material [37], as can be clearly seen in the SEM image in Figure 4.

PEO and bicomponent fibers had similar roughness parameters. Even though the diameters of fibers could influence roughness, an increase of 100 nm in the diameters of the bicomponent fibers did not seem to be enough to change the topography, at least at the studied scale given by the profilometer.

**Figure 5:**
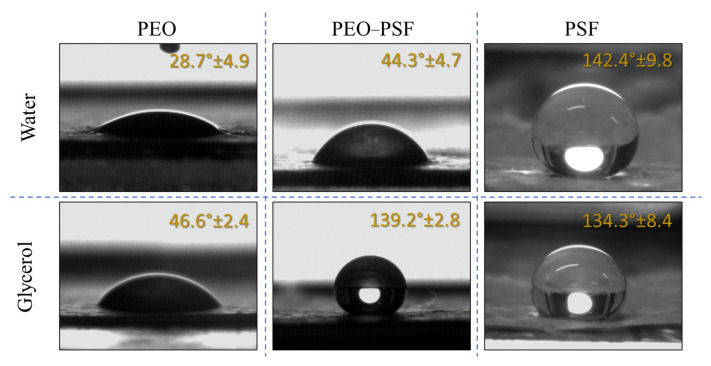
Images of test liquid drops and contact angles obtained on PEO, PEO-PSF, and PSF fibrillar materials fabricated by SBS.

The contact angle values were measured using two test liquids, water and glycerol, from the sessile drop method. Representative images of the liquid drops on the surface of the fibrillar materials obtained by SBS are shown in Figure 5.

The water contact angle measured in PSF-based fibrous materials with similar micro-constituents was previously reported for optimized SBS processing conditions. In that case, contact angles of 159.5° and 158.6° for water and glycerol, respectively, were obtained [8]. PSF fibers produced with a unoptimized system were 142.4° and 134.3° for water and glycerol, respectively. Due to variations in parameter production, surface morphologies in terms of diameter and rate of fibers were modified. Even though the contact angle changed, the material was still superhydrophobic [8,37,38].

On the other hand, the contact angle obtained for PEO fibers was similar to that obtained by other authors for the same material for glycerol but fabricated by electrospinning [39]. At this point, it must be considered that water can dissolve the PEO, so the contact angle reading can be doubtful.

Finally, the same study was conducted for materials made up of bicomponent fibers with a coaxial structure. As expected, the contact angle in water was low, 44.3°, due to PEO (hydrophilic) constituting the external part of the fibers and dissolving as water came in contact with the material [23]. However, bicomponent fibers showed a higher contact angle in comparison with PEO fibers. There are two possible reasons for this observation: the first one to consider is that a small fraction of PSF might be located on the surface, as would occur for the S/S fibers. The second reason is the dissolution of the PEO shell, making accessible to the test liquid the highly hydrophobic core PSF-rich phase of the fibers. In this sense, the high contact angle for the bicomponent material could be explained. The PSF fraction on the material’s surface supported the glycerol drop, as the Cassie–Baxter approximation explained for the PSF system [10].

**Figure 6:**
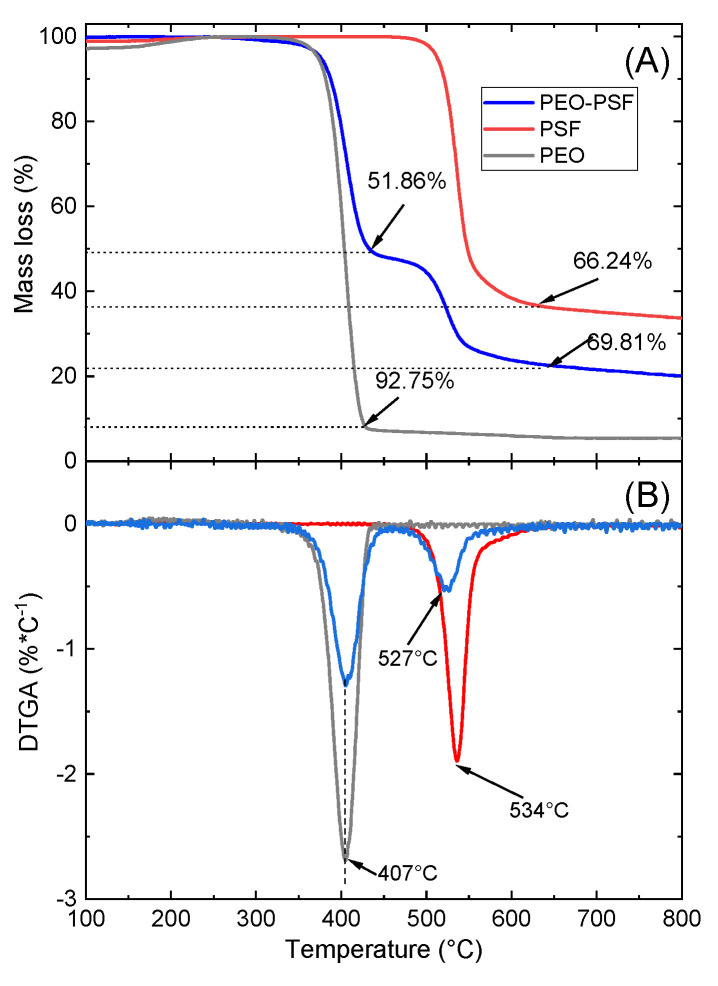
TGA (**A**) and DTGA (**B**) curves of PSF, PEO, and PEO-PSF fibrillar materials.

Thermogravimetric analysis (TGA) and differential thermogravimetry analysis (DTGA) were conducted to investigate the materials’ thermal behavior. In Figure 6, thermodegradation phenomena can be visualized through the TGA and DTGA curves. As can be seen, neat PSF and PEO fibers showed only one degradation process, with maximum rates at 534 °C and 407 °C, respectively. Moreover, under a nitrogen atmosphere, almost all PEO polymer was transformed at 800 °C into volatile components (92.75%), while for the PSF, 35% of the residues remained. These results are consistent with others obtained for PSF [40] and PEO [41,42] processed by other methods, showing that SBS does not cause any structural or morphological changes that influence the thermodegradation behavior of these polymers.

On the other hand, the thermodegradation behavior of the SBS bicomponent material PEO-PSF is also shown in Figure 6. As expected, two clear degradation steps were observed that could be assigned to the degradation processes of the two polymers. The first one was characterized by a maximum rate of degradation temperature at 407 °C, exactly at the same position as that obtained for the neat PEO, and the second one with a maximum rate of degradation located at 527 °C, slightly lower than the temperature found for the neat PSF. The structure formed with fibers without microconstituents and a fraction of PSF fibers with side-by-side structure could cause a decrease in degradation temperature of 10 °C.

The mechanical behavior of the fibrous materials usually depends on several factors, such as the intrinsic characteristics of the polymers (specific interactions, molecular weight), composition and internal morphology in the case of mixtures, and external morphology (size of microconstituents for example, fiber diameter, presence of defects and porosity) [39,43]. In this work, several of these factors were considered when comparing fibrous materials from neat polymers (PSF and PEO) and obtained by SBS with PSF-PEO bicomponent material also obtained by SBS.

**Figure 7:**
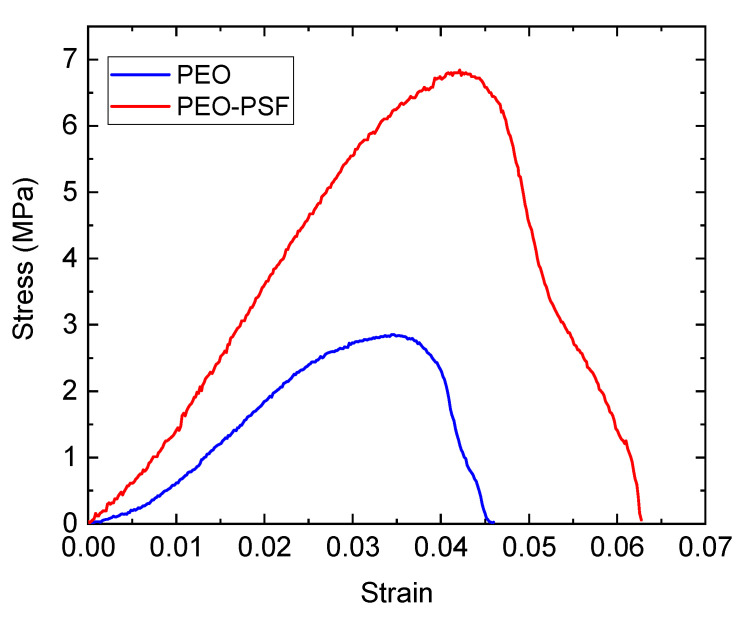
Significant stress–strain curves of fibrous materials: PEO and PEO-PSF.

Figure 7 shows the stress–strain curves for PEO and bicomponent PSF-PEO materials obtained by SBS. Tensile tests of SBS neat PSF materials could not be carried out because of a lack of mechanical consistency in the prepared specimens to be tested. The reason is that relatively short fibers of PSF can be obtained from SBS, avoiding the possibility of entanglements, thus allowing the required mechanical consistency. Moreover, PSF is a quite rigid and brittle material that, together with its presence in the form of thin fibers, must lead to even poorer mechanical consistency.

Pure PEO fibers fabricated by SBS showed Young’s modulus of 136.2 ± 45.2 MPa, a tensile strength of 2.7 ± 0.8 MPa, elongation at break of 4.2% ± 0.7, and porosity of 64.9%. Although the tensile strength of PEO-based materials obtained in this work by SBS was comparable to that of PEO fibrous materials fabricated by electrospinning (2.5 ± 0.5), Young’s modulus and strain at failure varied due to the electrical charge applied on electrospinning, which could modify the crystallinity of the material and consequently some mechanical properties as a tensile module [39].

For the bicomponent material, the following mechanical parameters were obtained: Young’s modulus of 183.76 ± 60.17 MPa, tensile strength of 6.6 ± 1.55 MPa, elongation at break of 6.7 ± 1.4, and porosity of 88.2%. Although the elastic module of nanofibers tends to decrease as the diameter of fibers increases for mono-component fibers [27], bicomponent fibers increase the tensile module compared with pure material despite the increasing diameter of the fibers. Indeed, tensile strength and elongation at break of the bicomponent fibers increase compared with the pure material. Conceivably, PSF fibers inside the PEO fibers acted as reinforcement material to increase the mechanical properties of the bicomponent material, as reported by Ma et al. [44], as contact between soft (PEO) and hard (PSF) materials regardless of core structure was centered or close to the wall of the shell structure. Indeed, higher rigidity near the fiber surface could result in a stiffer mat due to an increased stiffness at fiber-to-fiber junctions [45]. Another hypothesis is that the blend of solvents used in PEO dissolution could affect the mechanical properties of polymeric nanofiber due to the relaxation process generated by the evaporations rate [27,46].

## 4. Conclusions

Nonwoven mats of bicomponent fibers of Poly (ethylene oxide)—Polysulfone were fabricated by the solution blow spinning method and compared with parallel neat fibrillar materials. Bicomponent fibers exhibited fibrillar morphology without the presence of microconstituents with a core-shell structure. Although STEM and FTIR confirmed the PSF-core and PEO-shell structures, the core structure fraction was not centered and was located close to the shell wall. Indeed, FTIR spectra provided no evidence of miscibility between the core and shell materials. Thermal degradation of the bicomponent material was in two steps, the first corresponding to PEO, and the second to PSF material. The fraction of the core close to the shell wall and the roughness of the material promoted an increase in wettability when glycerol was tested due to the hydrophobic nature of PSF. Mechanical properties were improved due to the PSF-core fiber, which served as reinforcement material; additionally, PEO was used as the assisted formation of the PSF core in the form of fibers without microconstituents. The bicomponent material could even be used for filtration as a medical proposal for the hydrophobic and mechanical properties.

## Figures and Tables

**Table 1:** Roughness parameters obtained for PEO, PSF, and bicomponent fibrillar materials produced by SBS.

Sample	R_a_ [µm]	R_y_ [µm]	S_m_ [µm]
PEO	2.6 ± 0.4	16.8 ± 1.5	14.4 ± 1.8
PEO-PSF	3.1 ± 0.6	19.1 ± 3.2	15.9 ± 3.2
PSF	3.4 ± 0.8	15.6 ± 4.2	233.5 ± 35.8

## Data Availability

Not applicable.
